# A data-driven state identification method for intelligent control of the joint station export system

**DOI:** 10.1038/s41598-025-87283-2

**Published:** 2025-01-22

**Authors:** Guangli Xu, Yifu Wang, Zhihao Zhou, Yifeng Lu, Liangxue Cai

**Affiliations:** 1 School of Oil & Natural Gas Engineering, Southwest Petroleum University, Chengdu, 610500 Sichuan China; 2 Oil & Gas Fire Protection Key Laboratory of Sichuan Province, Chengdu, 610500 Sichuan China

**Keywords:** State identification, Parameter prediction, GWO, PSO, BP neural network, Mechanical engineering, Crude oil

## Abstract

As a necessary part of intelligent control of a joint station, the automatic identification of abnormal conditions and automatic adjustment of operation schemes need to judge the running state of the system. In this paper, a combination of Particle Swarm Optimization (PSO) and Gray Wolf Optimizer (GWO) is proposed to optimize the Backpropagation Neural Network (BP) model (PSO-GWO-BP) and a pressure drop prediction model for the joint station export system is established using PSO-GWO-BP. Compared with the traditional hydraulic calculation modified (THCM) models and other machine learning algorithms, the PSO-GWO-BP model has significant advantages in prediction accuracy. Based on the PSO-GWO-BP pressure drop prediction model, the determination method of state identification threshold is established, and a state identification method based on dynamic threshold is proposed, which realizes the intelligent identification of the system operation state by automatically adjusting the threshold. Through the analysis of the production and operation data of the joint station, the abnormal working conditions are successfully identified, and the effectiveness and accuracy of the method are verified. This method not only enhances the ability to discriminate abnormal working conditions but also adaptively adjusts the operation scheme, which effectively improves the intelligence level of the joint station export system.

## Introduction

The oil field joint station is gradually transitioning from the digital stage to the intelligent stage, driven by the rapid advancement of artificial intelligence technology^1,2^. The construction of an intelligent joint station will significantly improve production quality, accelerate production progress, and enhance safety control ability^3^. In the process of realizing the intelligence of a joint station, state identification is a key link. Its core objective is to carry out scientific and reasonable assessment based on process parameters and equipment status information obtained in real-time to provide strong support for subsequent control decisions. State identification covers the contents of single equipment state monitoring and process parameter state assessment, of which process parameter assessment is critical. Process parameter assessment through the essence of parameter prediction, the real-time monitoring values, and predicted values for comparison and analysis. To determine whether the parameters are in a normal range and to divide the normal and abnormal working area^4,5^, which lays the foundation for the subsequent abnormal working condition identification.

There are two primary methods for real-time detection of working condition changes. The first method involves directly using steady-state operation data to simultaneously detect and identify working conditions. The second method first determines whether an abnormal working condition has occurred before proceeding to identify the specific condition. Since steady-state operation data constitutes a large portion of actual production data, the first method requires concurrent abnormal detection and working condition identification, which has been proven to lower the efficiency of continuous condition detection and consume significant system resources. In contrast, the second method improves system efficiency in recognizing abnormal conditions. Once an abnormal condition is successfully identified, real-time data is routed to an intelligent processing module, where it is analyzed for specific abnormal condition judgment and appropriate handling. At present, there are fewer studies on the effective identification and judgment of operating conditions of the joint station export system, and previous research on the identification of the conditions mainly focuses on abnormal conditions, such as pipeline leakage^6^ and pump dumping, and abnormal conditions are directly investigated, and abnormal conditions are identified through the extraction and analysis of the signal features^7,8^, without distinguishing between normal and abnormal conditions and then carrying out specific condition identification.

The equipment of the joint station export system generally includes oil transfer pumps, heaters, pipelines, and various measuring instruments, involving parameters such as pressure drop, temperature, and flow rate. The temperature and flow rate of oil will cause pressure changes, and at the same time, the pressure drop has high sensitivity and strong adaptability^9^ in identifying abnormal working conditions such as pipeline leakage, pump failure, insufficient resistor, and screen blockage, which can be used as a diagnostic parameter for condition identification. Accurate prediction of process parameters^10^ is the key to identifying whether operation status is normal or not and is also a prerequisite for the intelligent construction of a joint station, which requires accurate prediction of pressure drop.

Pressure drop prediction generally adopts the traditional hydraulic calculation formula, and the commonly used Darcy formula calculation method is simple and intuitive. Due to the complexity of pipeline operating conditions, pipeline roughness and other parameters are difficult to measure in time, and there are more empirical coefficients in the Darcy formula. Numerous researchers have proposed many correction methods to calculate the pressure drop. Chen et al.^11^ corrected the pressure drop prediction model based on the experimental data of pressure gradients and liquid-holding rate. Zhan^12^ used the method of correcting the friction factor of the air–liquid interface with the calculation model of the pressure drop to establish a hydraulic calculation model. Liu et al.^13^ corrected the Levenberg–Marquardt (L-M) relationship equation with the experimental data and calculated the friction pressure drop of the air–water cyclonic flow in the horizontal pipe. Zhao et al.^14^ used the method of least squares polynomial correction and empirical formula coefficient correction to correct the hydraulic calculation formula for different types of wastewater pipelines in the oil field. Similarly, Zhang^15^ and Ye^16^ used the least squares method to correct the friction coefficients along the length of the oil pipeline. It can be seen that all these pressure drop prediction methods are corrected using experimental or field data to form a modified model for traditional hydraulic calculation.

With the development of machine learning technology, an increasing number of studies have focused on parameter prediction of oilfield process systems using deep learning and other data-driven models. Shin et al.^17^ used experimental friction pressure gradient data from gas–liquid two-phase flow to validate the accuracy of prediction of the Homogeneous Equilibrium Mixture Model (HEM) correlation friction pressure gradient model proposed by Garcia et al. Halali et al.^18^ proposed using a Radial Basis Function Neural Network (RBF-NN) model to predict pressure gradients. Zhang et al.^19^ proposed the use of Physics-Guided Neural Network (PGNN) to predict the pressure drop and liquid holding rate. Li et al.^20^ constructed a Chaotic Particle Swarm Optimization combined with Radial Basis Function Neural Network (CPSO-RBF) model to predict pipeline oil temperature and pressure. Shadloo^21^ utilized a Multilayer Perceptron Neural Network (MLPNN) to predict pressure drops of gas–liquid two-phase flow in horizontal pipelines. Faraji et al.^22^ optimized a multilayer perceptron using various optimization algorithms to predict two-phase flow friction pressure drops. The above research proves the applicability of neural networks in parameter prediction direction. Xiao et al.^23^ used the Whale Optimization Algorithm (WOA) to optimize BP to predict liquid holding in horizontal wet gas pipelines. Ma et al.^24^ used the PSO algorithm to optimize BP to achieve the prediction of the pressure drop of water coke slurry in pipeline transportation. Zhang et al.^25^ used Genetic Algorithm (GA) to optimize BP to develop a prediction model for the energy efficiency index of crude oil gathering and transportation systems. Shnain et al.^26^ used Levenberg–Marquardt BP, Bayesian Regularized BP and Scaled Conjugate Gradient BP to predict several parameters of water–oil two-phase flow. The above scholars used optimization algorithms to optimize the neural network weights and biases in order to avoid the BP from falling into the local optimal solution^27^, which improved the global search ability of the BP. However, a single optimization algorithm is limited in its ability to cope with complex problems, and it is difficult to fully explore the solution space; thus it cannot always ensure that a globally optimal solution is obtained. In recent years, the fusion of multiple optimization algorithms has emerged, and it is a feasible solution to further improve the optimization effect by combining different algorithms. For the optimization of BP, there are different algorithms for optimal tuning, such as WOA, GWO, PSO, and Pelican Optimization Algorithm (POA)^28^, etc., and the research can compare the established model with the optimization results of these models^29^. Considering the advantages of the GWO algorithm with strong global search capability and adaptivity^30^ and the PSO algorithm with strong local search capability, fast convergence, and few parameters, in this paper, we will use a combination of the PSO algorithm and the GWO algorithm to optimize the BP.

This paper proposes a data-driven state identification method to determine whether abnormal working conditions occur. The method predicts the key parameters of the export system, calculates the deviation between the predicted value and the real value, compares the deviation with the threshold value, and if the deviation exceeds the threshold value, it is recognized by the system as an abnormal working condition, so as to realize the state recognition. In this way, the system resource consumption of the working condition identification model can be effectively reduced, and its operation efficiency can be significantly improved. The main contributions of this paper are as follows:

1. A state identification method combining process parameter prediction and dynamic thresholding to discriminate normal or abnormal working conditions is proposed.
2. A PSO-GWO-BP model is proposed, which combines the advantages of PSO algorithm and GWO algorithm in optimizing the BP compared with the existing literature, sets the algorithm switching, and finally establishes a pressure drop prediction model for the joint station export system by using PSO-GWO-BP.
3. For the same training dataset, the traditional hydraulic correction model (THCM), PSO algorithm optimized BP (PSO-BP), GWO algorithm optimized BP (GWO-BP), POA algorithm optimized BP (POA-BP), Extreme Gradient Boosting (XGBoost), and the deep learning method convolutional neural network (CNN) pressure drop prediction models, and compare the PSO-GWO-BP model to these algorithms to ensure the highest prediction accuracy.
4. Parameter prediction is carried out using drastically changed field production and operation data as an example, and dynamic threshold judgment is used, and finally two working conditions are successfully identified as abnormal conditions.

This paper is organized as follows. First, in Sect. 2 “State identification method”, we establish the state identification method and elaborate on its development process and model description. Second, in Sect. 3 “Results and discussion”, we introduce the parameter determination of the pressure drop prediction model, the prediction results, the determination of the dynamic threshold, and verify the accuracy of the model through two cases of abnormal working conditions.

## State identification method

**Fig. 1:**
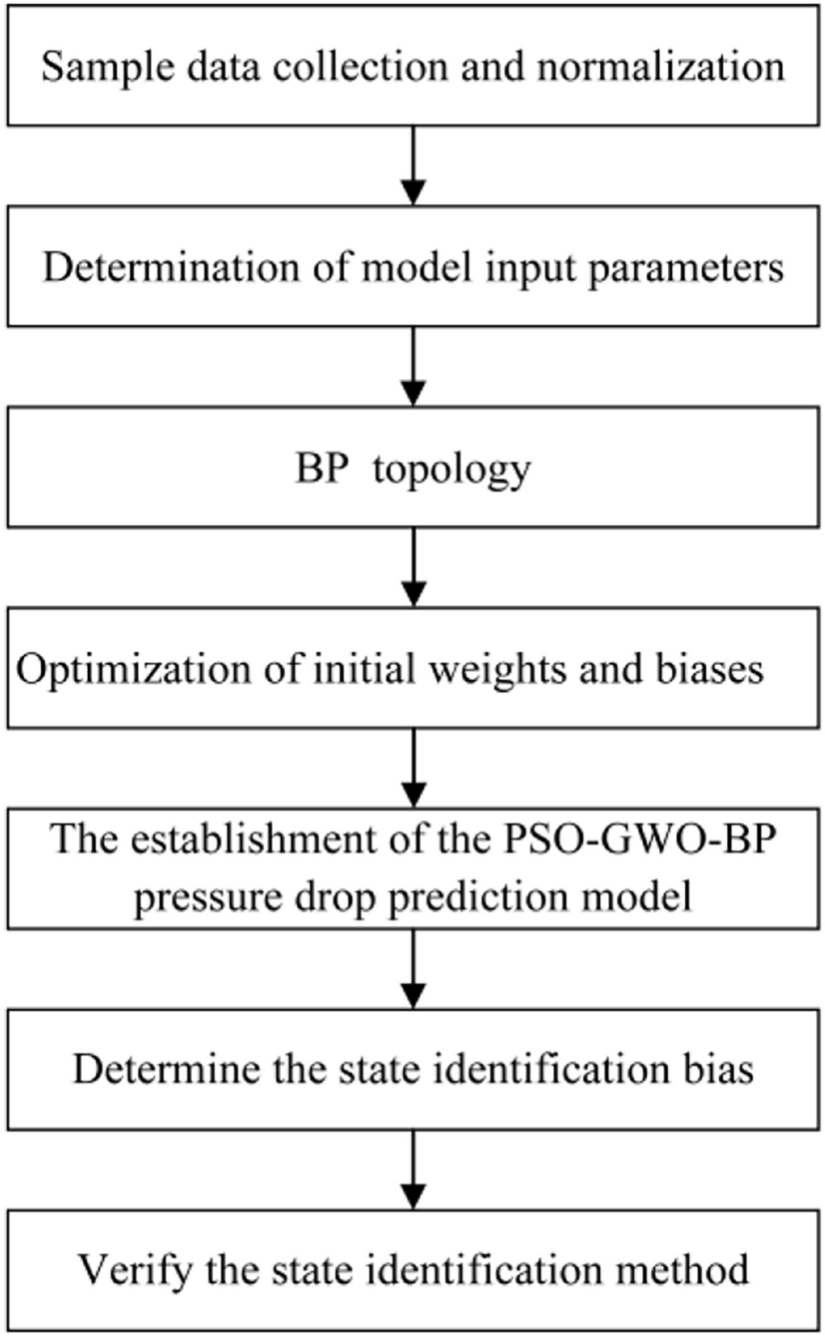
State identification method establishment flow.

The state identification method will be established through the process shown in Fig. 1, with the specific steps as follows: (1) Collect sample data to establish the model dataset and normalize the data; (2) Determine the input and output parameters of the model; (3) Define the topology of the BP; (4) Optimize the initial weights and biases using a combined optimization algorithm of PSO and GWO; (5) Establish the PSO-GWO-BP pressure drop prediction model; (6) Determine the state identification threshold; (7) Verify the state identification method.

### Sample data collection and normalization

The 12,000 sets of data from the operation of the export system at the LE joint station are used as the object of the study, including different months of the year, different flow rates, and different pressure drops. The data contains starting pressure, starting temperature, ending pressure, ending temperature, oil density, and flow rate. The outlet of the export system is the inlet of the pipeline, and the downstream is the YL station, which is responsible for receiving the oil products from the LE joint station and is the outlet of the pipeline. The design pressure of the pipeline is 5 MPa, and the whole line adopts the heating and confinement conveying process, the length of the pipeline is 22 km, the inner diameter is 130 mm, the depth of the pipeline is 1.5 m, and there is a heat preservation layer along the way, so the temperature drop is small. The flow rate is 80 ~ 100 m^3^/h, the inlet oil temperature is 42 ~ 46 °C, and the outlet oil temperature is 38 ~ 40 °C.

In order to facilitate the acceleration of convergence, eliminate the influence of feature scale, and improve the model training effect, the data are normalized. The original data are normalized to the [0,1] interval, and the normalization formula is shown in Eq. (1) ^31^.

1 $$X\prime = \frac{{X - X_{\min } }}{{X_{\max } - X_{\min } }}$$

where $X\prime$ is the normalized data, *X* is the original data, and *X*_min_ and *X*_max_ are the minimum and maximum values in the sample data, respectively.

### Determination of model input parameters

Before BP training, the input parameters and output parameters of the model need to be determined. First, the input parameters of the model are analyzed by correlation analysis. Correlation analysis can identify the features that have the greatest impact on the prediction results, so as to optimize the input feature set and improve the prediction performance of the model.

In this paper, the Pearson correlation coefficient in correlation analysis is selected, and its calculation formula is shown in Eq. (2) ^32^.2

where *r* is the correlation coefficient, *x* and *y* are two variables, and *n* is the sample size.

The factors influencing pressure drop in oil pipelines include pipe diameter, pipe length, pipe roughness, flow regime of the oil, gravitational acceleration, oil density, oil viscosity, starting pressure, flow rate, starting temperature, and ending temperature. For a specific pipeline, the pipe diameter, length, roughness, flow regime, and gravitational acceleration are constant values, and both viscosity and density are functions of temperature. Therefore, only density is selected for consideration in the correlation analysis. Thus, the parameters chosen for correlation analysis are starting pressure P_s_, flow rate Q, starting temperature T_s_, ending temperature T_e_, oil density *ρ*, and pressure drop ΔP.

### BP topology

The BP^33^ is a widely applied multilayer feedforward neural network that utilizes the error backpropagation algorithm for training. The core idea of the BP is to continuously adjust network weights and biases to minimize the error between predicted and actual outputs, achieving nonlinear mapping and data association for complex variables.

**Fig. 2:**
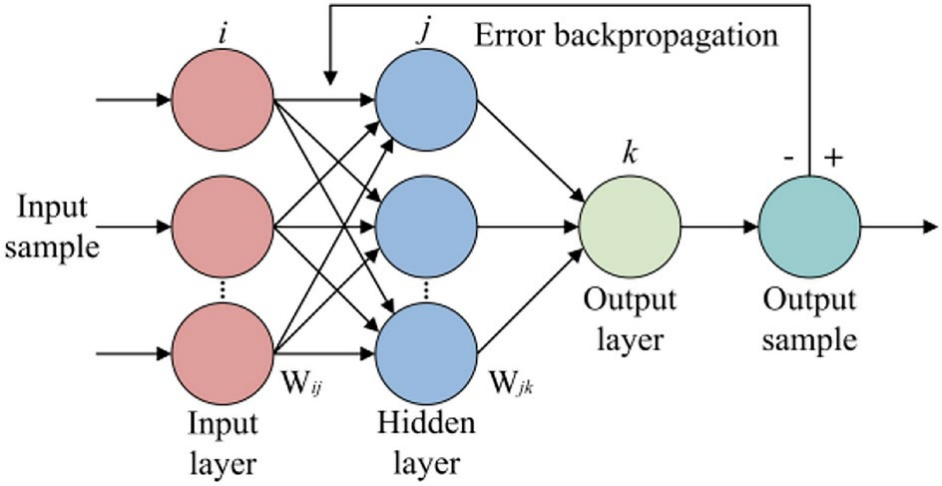
BP structure: including input, output, input layer, hidden layer, output layer and error backpropagation.

A BP typically consists of three parts: The input layer, hidden layer, and output layer. In operation, the input layer receives external input data, with each neuron corresponding to an input variable. The hidden layer is responsible for extracting features and patterns from the input data; it may consist of one or more layers, with each hidden layer neuron fully connected to neurons in the preceding and subsequent layers. The output layer generates prediction results, with each neuron corresponding to an output variable. The structure of the BP is shown in Fig. 2.

In a BP, the network topology must be determined before training, and this study uses a single hidden layer structure. The number of hidden layer nodes directly impacts the model’s complexity and performance, making it essential to select an appropriate number of hidden nodes to construct an effective neural network. The optimal number of hidden nodes is typically determined through experience and experimentation. In this study, the range for the number of hidden nodes is established using Eq. (3) ^34^, and the optimal number is determined by trial and evaluation.

3 $$m = \sqrt {n + l} + h$$

where *m* is the number of hidden layer nodes, *n* is the number of input layer nodes, *l* is the number of output layer nodes, and *h* is a constant between 1 and 10.

### Optimization of initial weights and biases

The BP is essentially a gradient descent method, which has notable. Advantages such as adaptability, self-learning, high fault tolerance, and strong fitting ability. However, the BP can produce significant result differences depending on the choice of initial weights and biases. It is prone to issues such as getting stuck in local minima, long training times, and the vanishing gradient problem^35^. Therefore, to address the shortcomings of the BP, the PSO algorithm, with its strong local search capability, fast convergence, and fewer parameters, and the GWO algorithm, with its strong global search ability and adaptability, are combined to optimize the BP weights and biases. A phase-based strategy is introduced to dynamically update the positions of the grey wolves to find the optimal weights and biases. Below, we introduce the PSO algorithm and the GWO algorithm and explain the parameter selection or update methods.

Kennedy and Eberhart proposed the PSO^36^ algorithm in 1995. The inspiration for PSO comes from the foraging behavior of birds. By simulating the movement of individuals (particles) in a population and information sharing among them, PSO searches for the optimal solution to a problem. It is a heuristic algorithm that incorporates individual improvement, population cooperation, and competition mechanisms. In the PSO algorithm, each solution is considered a particle in the search space. The particle moves in the multi-dimensional space and gradually approaches the optimal solution. Particles adjust their positions and velocities based on both their own and the group’s experiences. The goal of the algorithm is to find the solution that optimizes the objective function by the collaborative work of particles in the population. The updates of the velocity and position of the *i*-th particle in the *j*-th dimension of the decision space are given by Eqs. (4) and (5)^36^.

4 $$v_{ij}^{k + 1} (t + 1) = \omega v_{ij}^{k} (t) + c_{1} r_{1} (p_{ij}^{k} (t) - x_{ij}^{k} (t)) + c_{2} r_{2} (p_{gj}^{k} (t) - x_{ij}^{k} (t))$$

5 $$x_{ij}^{k + 1} (t + 1) = x_{ij}^{k} (t) + v_{ij}^{k + 1} (t + 1)$$

where $v_{ij}^{k} (t)$ is the velocity of the *i*-th particle in the *j*-th dimension at the *k*-th iteration, and $x_{ij}^{k} (t)$ is the position of the *i*-th particle in the* j*-th dimension at the *k*-th iteration. $\omega$ is the inertia weight, which controls the inertia of the particle’s movement and determines the tendency of the particle to maintain its current velocity. *c*_1_ and *c*_2_ are learning factors that represent the acceleration weights for the particle to follow its own best historical solution ($p_{ij}^{k} (t)$) and the global best solution ($p_{gj}^{k} (t)$), respectively. *r*_1_ and *r*_2_ are random numbers between 0 and 1, which increase the randomness of the particle’s movement.

When using the PSO algorithm, the setting of parameters has a significant impact on the optimization results and convergence speed. In this study, the main parameters of the PSO algorithm, *c*_1_, *c*_2_ and $\omega$, are initially set to 1.5, 1.5, and 0.9, respectively. During the iteration, a dynamic adjustment method is adopted to update the parameters. The specific updates are shown in Eqs. (6) to (11).

If the current best fitness is smaller than the previous round’s best fitness, *c*_1_ and *c*_2_ are increased to allow the search process to place more emphasis on personal experience (*c*_1_) and group experience (*c*_2_) in order to accelerate the search process.

6 $$c_{{{\text{1new}}}} = c_{1} + f_{s}$$

7 $$c_{{{\text{2new}}}} = c_{2} + f_{s}$$

where *c*_1new_ and *c*_2new_ are the updated values of *c*_1_ and *c*_2_, respectively; *f*_*s*_ is the adjustment factor, set to 0.1.

When the current best fitness is greater than the previous round’s best fitness, *c*_1_ and *c*_2_ are reduced to decrease the search intensity, thereby avoiding excessive exploration. This encourages the algorithm to focus more on local search, which is achieved through a decay factor.

8 $$c_{{{\text{1new}}}} = c_{1} - f_{d}$$

9 $$c_{{{\text{1new}}}} = c_{1} - f_{d}$$

where *f*_*d*_ is the decay factor, which is set to 0.01.

10 $$\omega_{{{\text{new}}}} = \omega - \frac{{0.5n_{i} }}{{n_{s} - n_{p} }}$$

11 $$n_{p} = n_{s} \cdot p$$

where *n*_*p*_ is the number of iterations for switching from GWO to PSO; *n*_*i*_ is the number of current PSO algorithm iterations; *n*_*s*_ is the maximum number of iterations; $\omega_{{{\text{new}}}}$ is the updated value of $\omega$; and *p* is the post stage value.

GWO^37^ is a swarm intelligence optimization algorithm that simulates the hunting behavior of grey wolf packs, with strong global search capabilities. The main steps of the GWO algorithm include four parts: Hunting, encircling, searching for prey, and attacking the prey. In the GWO algorithm, the grey wolves in the swarm are divided into four levels: The *α* wolf leads the group and makes decisions and commands actions; the *β* wolf assists the *α* wolf by providing feedback and suggestions; the *δ* wolf listens to the commands of the *α* and *β* wolves and is responsible for protection; and the ordinary wolves follow the guidance of the previous three to hunt.

Based on the mathematical model of grey wolf hunting, the Eqs. (12) and (13) can be derived.

12 $$\vec{D} = \left| {\vec{C} \cdot \vec{X}_{p} - \vec{X}(t)} \right|$$

13

where $\vec{X}(t)$ is the position of the current grey wolf; $\vec{X}_{p}$ is the position of the prey; $\vec{D}$ is the distance between the current grey wolf and the prey; $\vec{A}$ and $\vec{C}$ are two control vectors, which are randomly generated to control the movement direction and distance of the grey wolf. The calculation methods for $\vec{A}$ and $\vec{C}$ are given by the Eqs. (14) and (15).

14 $$A = 2a \cdot r_{1} - a,a = 2 - \frac{2t}{{T_{\max } }}$$

15

where *a* is the convergence factor, which decreases linearly from 2 to 0 as the number of iterations increases; *r*_1_ and *r*_2_ are random vectors within the range [0,1]; *T*_max_ is the maximum number of iterations.

GWO tracks the positions of *α*, *β,* and *δ* of the three optimal gray wolves in the population, and updates the positions of ordinary wolves, as shown in Eqs. (16) to (19).16


17



18



19


where $\vec{X}_{\alpha }$, $\vec{X}_{\beta }$,and $\vec{X}_{\delta }$ are the positions of the best individuals of the *α*, *β*, and *δ* wolves, respectively, at the current iteration. $\vec{X}_{1}$、$\vec{X}_{2}$、$\vec{X}_{3}$ represents the positions of the other wolves in the group. $\vec{X}(t + 1)$ represents the positions of the other wolves in the group.

In the GWO algorithm, the number of gray wolves and the maximum number of iterations are two important parameters that directly affect the algorithm’s performance and computational efficiency. To determine the optimal number of gray wolves and the maximum evolution iterations, this study simulates different combinations of gray wolf population size and maximum iteration counts. The combination that yields the smallest error is selected as the optimal parameter set.

### The establishment of the PSO-GWO-BP pressure drop prediction model

The specific implementation of optimizing the weights and biases of the BP using the hybrid PSO and GWO algorithm is as follows: Set the parameter post stage to divide the optimization process into two stages: the early stage and the later stage. In the early stage, GWO is used for global search. The grey wolf optimization simulates the hunting behavior of wolf packs, utilizing the best solutions from the *α*, *β*, and *δ* wolves to guide the movement of other individuals and explore the search space. In the later stage, PSO is employed. During the PSO phase, each individual (corresponding to each wolf in GWO) has a velocity vector. The position of the individual is no longer dependent on the average position of *α*, *β*, and *δ* wolves but updated by the PSO velocity formula, allowing for a more precise local search and accelerating convergence towards the global optimal solution.

**Fig. 3:**
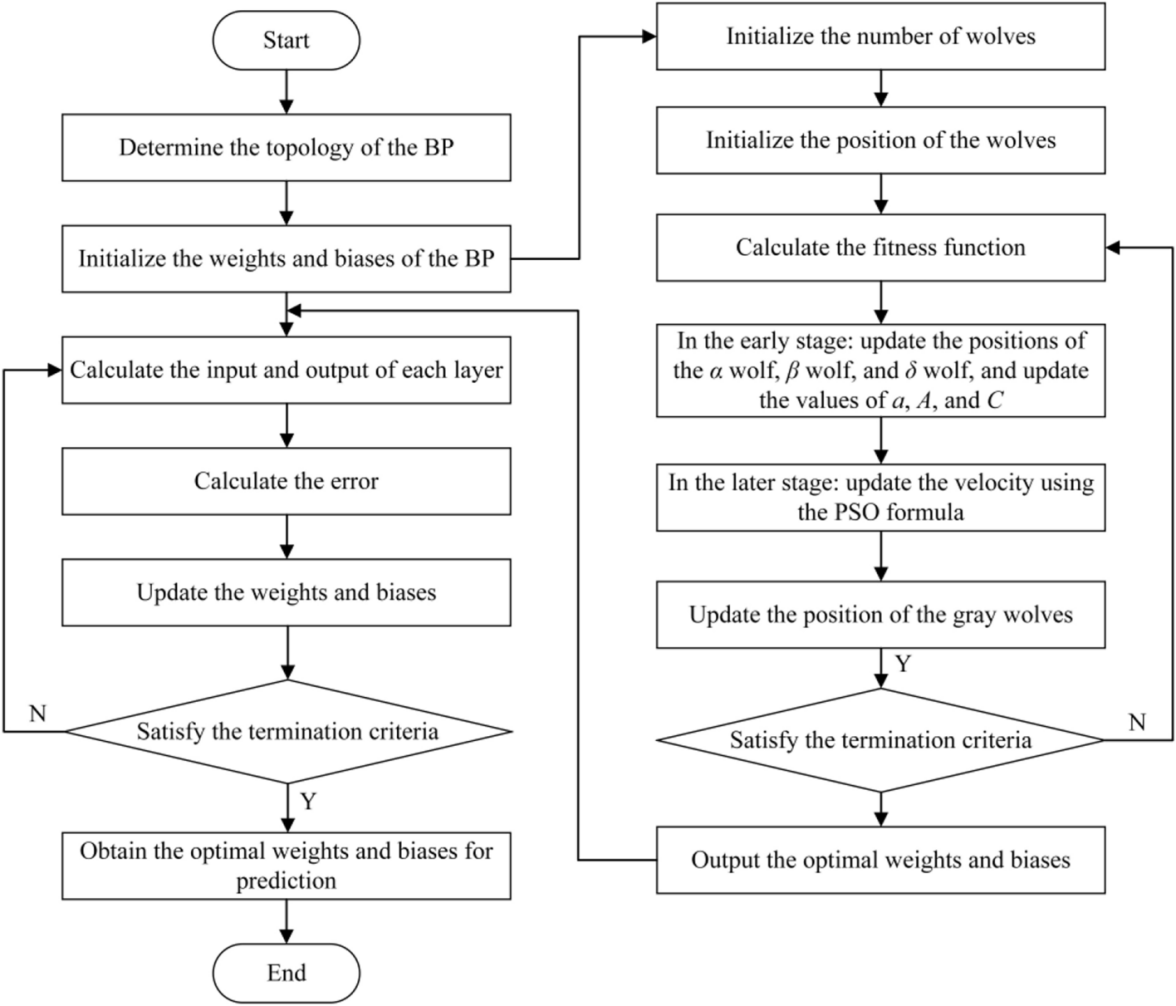
PSO-GWO-BP algorithm process.

The PSO-GWO-BP model process is shown in Fig. 3. The specific process is as follows:

- Step 1: Initialization: Initialize each grey wolf and its position in the GWO population. Also, initialize the weights and biases of the BP. Set the topology of the BP, including the number of neurons in the input layer, hidden layer, and output layer.
- Step 2: Evaluate individual fitness: Perform forward propagation and error calculation on the weights and biases of each grey wolf using the BP, obtaining the fitness of each individual.
- Step 3: Update wolf positions: Update the positions of the grey wolves based on the GWO rules. In the early stage, use the GWO wolf pack update rule, and in the later stage, use the PSO update rule to search for the optimal BP weights and biases.
- Step 4: Training: Use the optimized weights and biases obtained from PSO-GWO as input to the BP. The BP algorithm will further adjust the weights and biases through backpropagation to refine the search results.
- Step 5: Iterative updates: Repeat the PSO-GWO update rules, performing global search iteratively until the stopping criteria are met (e.g., error convergence or reaching the maximum number of iterations).
- Step 6: Output the optimal weights and biases: After training with the PSO-GWO-BP algorithm, output the final optimized weights and biases of the BP, which can then be used for testing or actual prediction.

To assess the reliability and accuracy of the model, evaluation is required. Common evaluation metrics include the coefficient of determination (R^2^), root mean square error (RMSE), and mean absolute error (MAE). Among these, the closer the R^2^ value is to 1, the better the predictive performance of the model. The closer the RMSE and MAE values are to 0, the closer the predicted values are to the actual values, indicating higher prediction accuracy of the model.

### Method for determining the state identification threshold

In the state identification of the intelligent control of a joint station export system, it is an efficient method to distinguish normal and abnormal conditions by using a threshold. By analyzing the deviation between the predicted and measured values, the normal operating range of the system is determined. When the deviation exceeds the set threshold, it indicates a potential abnormality in the system. The specific process is as follows:

1. Collect historical data from the system under normal operating conditions and calculate the mean and standard deviation of the deviation between the predicted and measured values under different operating conditions (assuming that the deviation follows a normal distribution, with a mean of *μ* and a standard deviation of *σ*, the deviation range should fall within [0, *μ* + 3*σ*]);
2. Set the threshold based on the allowable deviation range of the process, with the threshold range set in this paper as [0, *μ* + 3*σ*];
3. During real-time monitoring, compare the absolute value of the deviation with the threshold. If the deviation exceeds the threshold consecutively, it is identified as an abnormal state.

However, due to the variability in the production output of the joint station export system and the significant changes in system pressure drop with flow rate, a single threshold may not accurately distinguish between normal and abnormal states. To address this, this study employs prediction models to estimate the pressure drop under varying flow rates, calculate the corresponding deviation mean and standard deviation, and establish a dynamic threshold adjustment model. The calculation formula is shown in Eq. (20). When the absolute deviation between the predicted and measured values exceeds the dynamic threshold, the system is identified as abnormal.

20 $$\Delta {\text{P}}_{{\text{b}}} = \mu_{{\text{e}}} + k \cdot \sigma_{{\text{e}}}$$

where $\Delta {\text{P}}_{{\text{b}}}$ is the bias, MPa; *μ* is the mean of the deviation, MPa; *k* is the dynamic adjustment factor, which can take values of 1, 2, or 3; *σ* is the standard deviation of the deviation, MPa.

In the field application of intelligent control for the joint station export system , a sliding window method is employed to adapt to dynamic system changes and enhance the accuracy of state identification. This method uses a fixed-size window that retains the most recent data. As new data is added, the oldest data is removed, ensuring a consistent window size^38^. The mean and standard deviation of the threshold are dynamically recalculated within the window, allowing real-time bias adjustment. This approach effectively responds to system fluctuations, overcoming the limitations of fixed thresholds and significantly improving the accuracy of distinguishing between normal and abnormal operating conditions.

## Results and discussion

### PSO-GWO-BP model parameter determination and iteration results

(1) Model input.

**Fig. 4:**
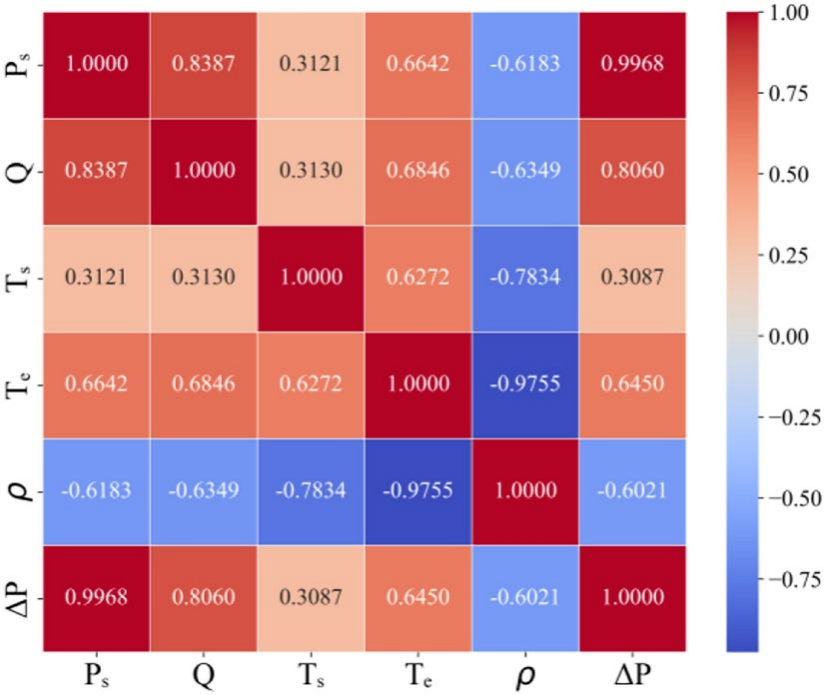
Correlation analysis matrix.

Positive and negative values in the correlation analysis represent positive and negative correlation, respectively. A correlation coefficient closer to 1 indicates a stronger correlation. Figure 4 shows the correlation analysis matrix for starting pressure P_s_, flow rate Q, starting temperature T_s_, ending temperature T_e_, density *ρ*, and pressure drop ΔP. From Fig. 4, the starting pressure, flow rate, and ending temperature exhibit strong positive correlations with the pressure drop, with correlation coefficients of 0.9968, 0.806, and 0.645, respectively. In contrast, the density shows a strong negative correlation with the pressure drop, with a correlation coefficient of -0.602. Based on these findings, the input parameters selected for the pressure drop prediction model are the starting pressure, flow rate, ending temperature, and density, while the output parameter is the pressure drop.

(2) Hidden layer nodes and learning rate.

**Fig. 5:**
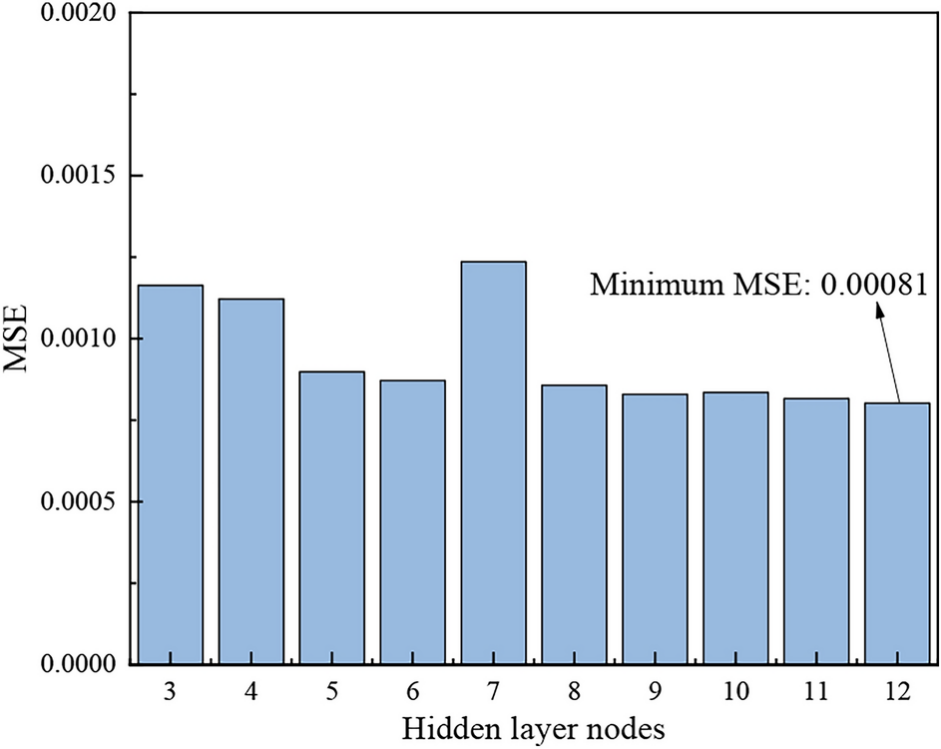
MSE change with hidden layer nodes diagram.

Based on the calculation from Eq. (3), the value of *m* ranges from 3 to 12. These values were substituted into the prediction model to simulate the performance of different hidden layer nodes. As can be seen from Fig. 5, the optimal number of hidden layer nodes is 12, which was therefore selected for the neural network configuration.

**Fig. 6:**
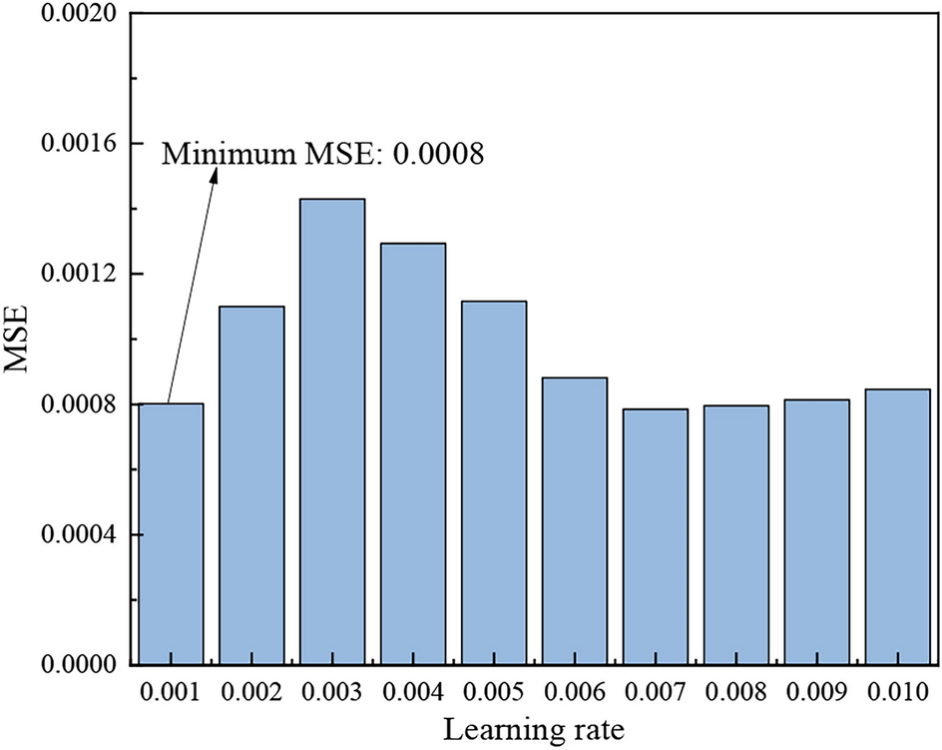
MSE change with Learning rate diagram.

In general, the learning rate is usually set to a small value, as an excessively large learning rate can lead to model divergence, while an overly small learning rate slows the convergence process. In this paper, the learning rate was set in the range of 0.001 to 0.01, with a step size of 0.001, for simulation experiments. As shown in Fig. 6, the MSE reaches its minimum when the learning rate is 0.001. Therefore, the optimal learning rate for the model is determined to be 0.001.

(3) PSO-GWO model parameter settings.

The weights and biases of the BP are treated as optimization variables in the PSO-GWO algorithm, with the MSE of the training and testing samples serving as the fitness function. The smaller value of the fitness function indicates that the training is more accurate and the prediction accuracy of the model is higher. The number of training times for BP learning is set to 50, the learning rate is 0.001, and the minimum error of the training objective is 0.0001, and the data are divided into a training set and a test set in the ratio of 8:2.

**Fig. 7:**
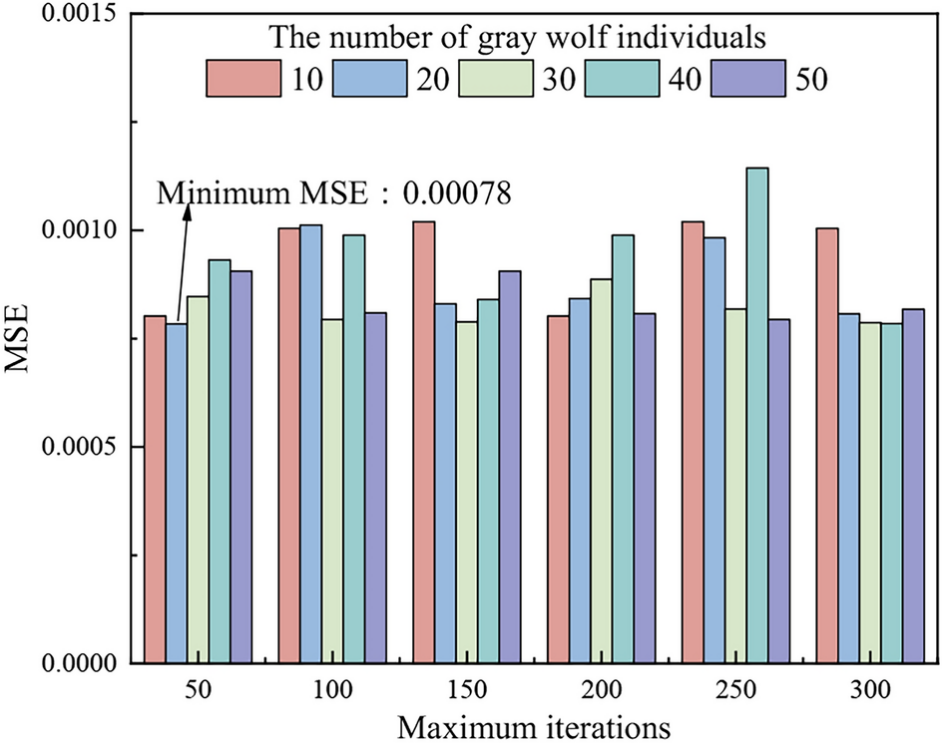
MSE changing with parameters of GWO diagram.

In the PSO-GWO algorithm, the number of gray wolf individuals and the maximum number of iterations are two key parameters that directly affect the performance and computational efficiency of the algorithm. To determine their optimal values, the number of gray wolf individuals was varied from 10 to 50 (step size: 10), and the maximum number of iterations was set between 50 and 300 (step size: 50). As shown in Fig. 7, increasing these parameters does not lead to a significant reduction in MSE. The minimum MSE is achieved with 20 gray wolf individuals and 50 iterations. Therefore, the optimal configuration is set as 20 gray wolf individuals and 50 iterations to optimize the weights and biases of the BP.

**Fig. 8:**
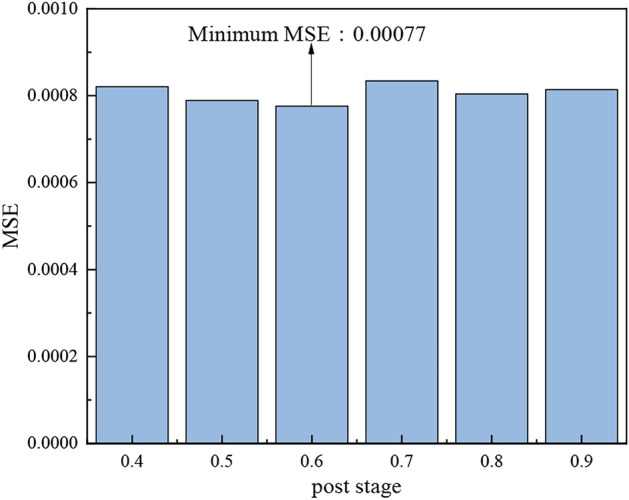
MSE changing with post stage.

In PSO-GWO optimization, the GWO algorithm is used in the early stage to carry out a global search and find the global optimal solution; in the later stage, it is switched to the PSO stage, which gradually reduces the search range and carries out local search and fine optimization. Therefore, when to switch has a great impact on the accuracy of the model. In this paper, the switching range is 0.4 ~ 0.9 and the step size is 0.1, and the model optimization is completed after simulation calculation. As shown in the Fig. 8, the MSE is the smallest when the post stage is 0.6, which is finally selected.

(4) Iteration results.

**Fig. 9:**
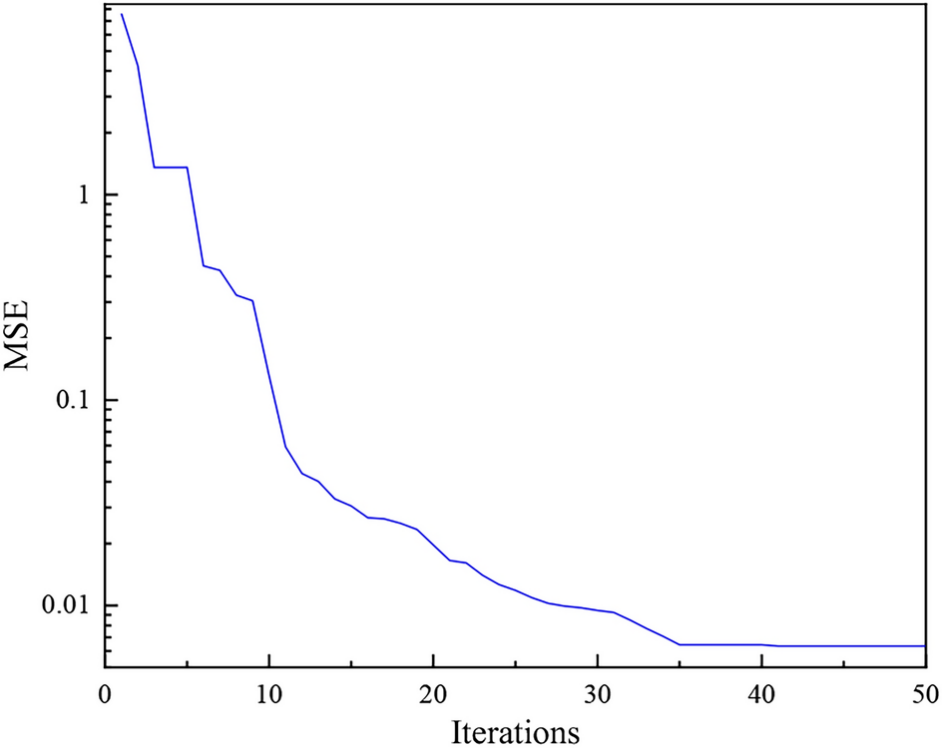
Change in cost function during iteration.

Substituting the sample data into the PSO-GWO-BP prediction model for simulation, the cost function changes are shown in Fig. 9, and the weights and biases of PSO-GWO optimization are basically stable after 50 iterations.

**Fig. 10:**
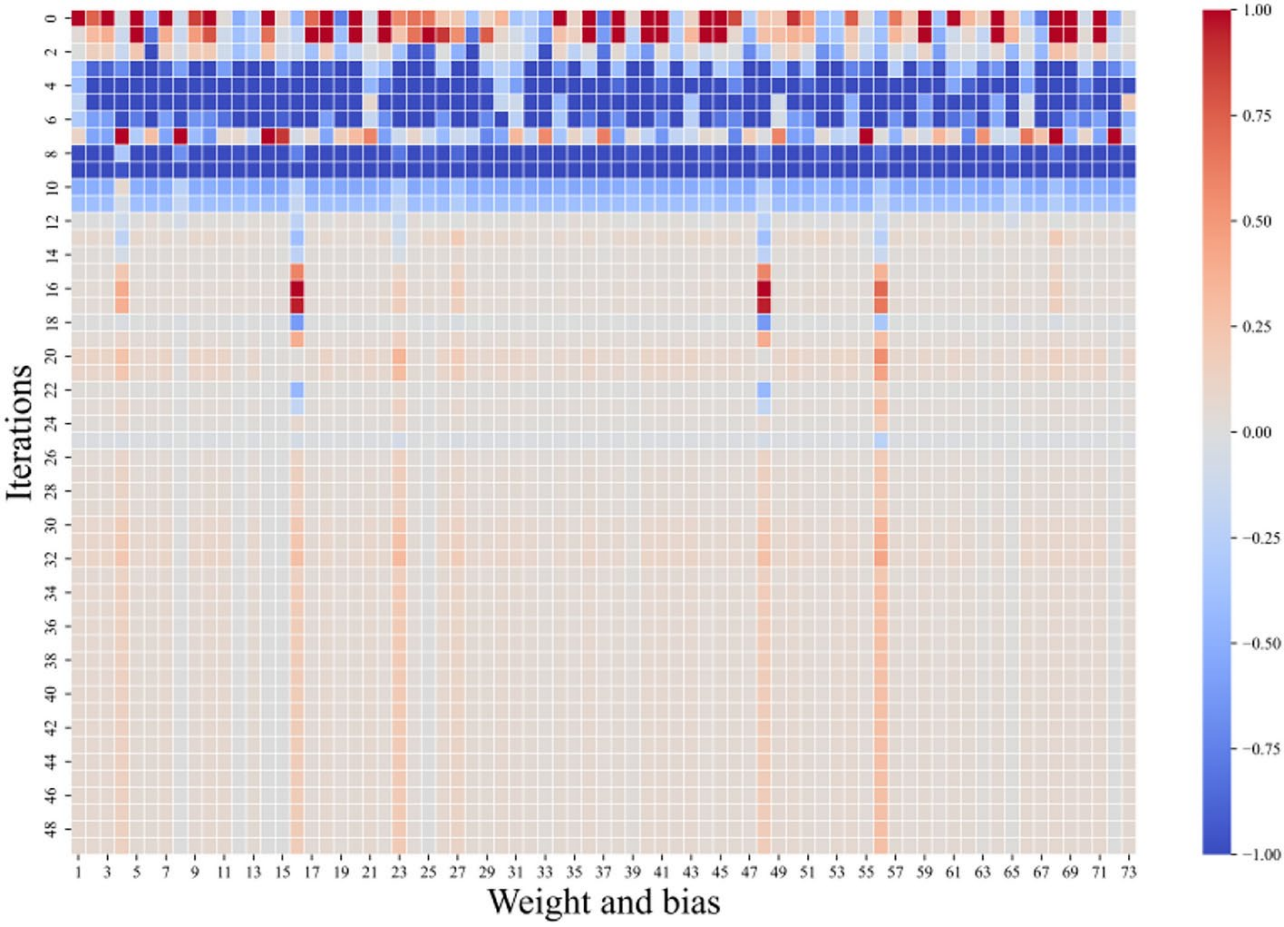
Heat map of weight and bias changes.

The weights and biases were varied as shown in the heat map presented in Fig. 10. The 73 variables on the horizontal axis are the weights and bias parameters from the input layer to the hidden layer (4 × 12 + 12) and the hidden layer to the output layer parameters (12 + 1). The vertical axis is the variation over 50 iterations. Among them, the optimal weights and biases are shown in Table 1.

**Table 1.** Optimal weights and bias of PSO-GWO-BP model.

Input weight				b_1_	Layer weight	b_2_
Input1	Input2	Input3	Input4			
0.0679	0.0535	0.0021	0.0402	0.0597	0.0567	0.0607
0.0675	0.0014	0.0653	0.0521	0.0472	0.0680	
0.0669	0.0046	0.1094	0.0175	0.0188	0.0497	
0.1443	0.1765	0.0494	0.0674	0.0473	0.0350	
0.0538	0.0366	0.0494	0.0682	0.0090	0.0676	
0.0533	0.0678	0.0641	0.0595	0.0661	0.0626	
0.0678	0.0392	0.0620	0.0694	0.0522	0.0317	
0.0030	0.0478	0.0537	0.0454	0.0680	0.2776	
0.0598	0.0676	0.0264	0.0476	0.0583	0.0508	
0.0693	0.0452	0.0528	0.0527	0.0594	0.0366	
0.0662	0.2008	0.0386	0.0386	0.0676	0.0611	
0.0036	0.0373	0.0703	0.1745	0.0014	0.0389	

### Comparison and analysis of prediction models

In order to compare the accuracy of the PSO-GWO-BP prediction model, the THCM, PSO-BP, GWO-BP, POA-BP, XGBoost, and CNN models were also developed.

**Fig. 11:**
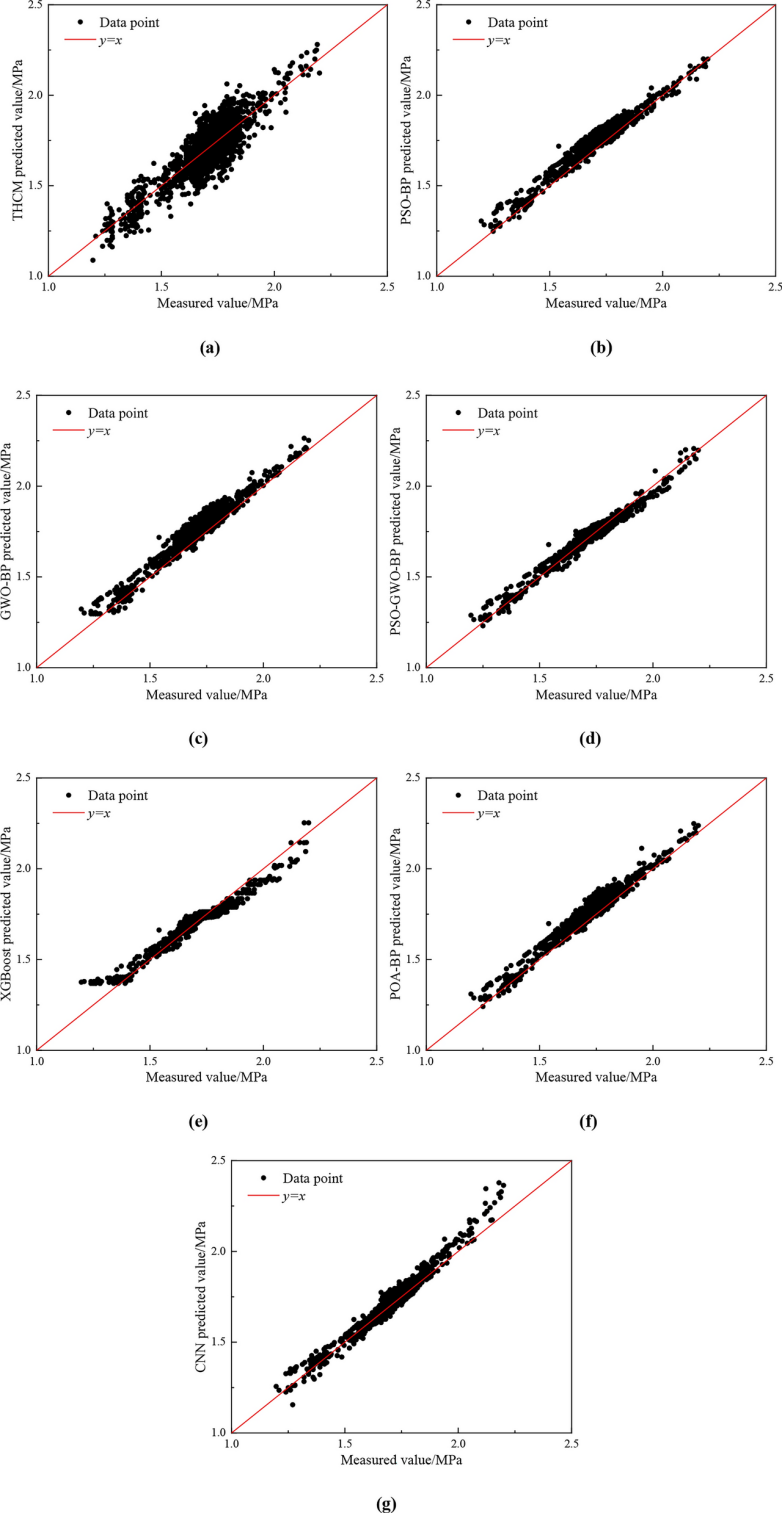
Comparison between predicted values and measured values of different models: **(a)** THCM model; **(b)** PSO-BP model; **(c)** GWO-BP model; **(d)** PSO-GWO-BP model; **(e)** XGBoost model; **(f)** POA-BP model; **(g)** CNN model.

As shown in Fig. 11, the predicted values of various models, including the THCM model (Fig. 11(a)), PSO-BP model (Fig. 11(b)), GWO-BP model (Fig. 11(c)), PSO-GWO-BP model (Fig. 11(d)), POA-BP model (Fig. 11(e)), XGBoost model (Fig. 11(f)), and CNN model (Fig. 11(g)), are compared with the measured values. From Fig. 11, it is evident that the THCM model performs poorly, particularly when the pressure drop is high. This is due to the least squares method used in parametric regression, which minimizes the squared difference between predicted and actual values. As a result, the model is highly sensitive to outliers, leading to inaccuracies in predicting extreme pressure drop values. In contrast, all other models show good performance with a high degree of fit. Notably, the PSO-GWO-BP model delivers accurate pressure drop predictions with minimal error compared to the measured values, demonstrating its clear advantage in addressing this prediction problem.

Table 2 presents the evaluation metrics for each model, accompanied by a scoring mechanism based on these metrics. A higher R^2^, lower RMSE and RAE, and higher score indicate better prediction performance. From the table, it is evident that the THCM model performs poorly in pressure drop prediction, with a low R^2^ of 0.8356 and high RMSE, and MAE, demonstrating poor model fit. In contrast, the machine learning models (PSO-BP, GWO-BP, PSO-GWO-BP, POA-BP, XGBoost, and CNN) all show good performance, significantly reducing RMSE and MAE, and achieving R^2^ values greater than 0.91, indicating substantial improvements in prediction accuracy. Among these, the PSO-GWO-BP model stands out with the highest R^2^ of 0.9877 and the top score of 21. CNN and XGBoost follow, with scores of 17 and 16, ranking second and third, respectively. The PSO-GWO-BP model outperforms the THCM, PSO-BP, GWO-BP, POA-BP, XGBoost, and CNN models, with R^2^ improvements of 18.20%, 7.57%, 9.03%, 6.25%, 5.34%, and 4.65%, respectively. In conclusion, the PSO-GWO-BP model demonstrates superior performance and high reliability in pressure drop prediction.

**Table 2.** Scoring results based on test set evaluation metrics.

No	Model	Test results			Score			Total score	Ranking
		RMSE(MPa)	MAE(MPa)	R^2^	RMSE	MAE	R^2^		
1	THCM	0.0726	0.0577	0.8356	1	1	1	3	7
2	PSO-BP	0.0512	0.0474	0.9182	3	2	3	8	5
3	GWO-BP	0.0549	0.0458	0.9059	2	3	2	7	6
4	PSO-GWO-BP	0.0304	0.0236	0.9877	7	7	7	21	1
5	POA-BP	0.0475	0.0407	0.9296	4	4	4	12	4
6	XGBoost	0.0447	0.0294	0.9376	5	6	5	16	3
7	CNN	0.0425	0.0321	0.9438	6	5	6	17	2

### Determination of the state identification threshold

In this paper, based on the flow range of the training model, a flow interval of 80–99 m^3^/h is selected. For each flow value, 3000 sets of data are extracted from the historical production data, and the mean and standard deviation of the deviation between the measured and predicted values are calculated. With reference to the previous analysis, the deviation range is determined as [0, *μ* + 3*σ*], with a dynamic adjustment factor of *k* = 3 to ensure that the model can accurately capture the state changes under different flow rates. Table 3 shows the results of threshold calculations under different flow rates, with the maximum threshold value of 0.0847 MPa and the minimum threshold value of 0.0303 MPa.

**Table 3.** Threshold calculation for state identification at different flow rates.

Q(m^3^·h^-1^)	*μ*_e_(MPa)	*σ*_e_(MPa)	ΔP_b_(MPa)	Q(m^3^·h^-1^)	*μ*_e_(MPa)	*σ*_e_(MPa)	ΔP_b_(MPa)
80.0196	0.0288	0.0186	0.0847	90.0092	0.0144	0.0095	0.0428
80.8728	0.0280	0.0188	0.0844	90.9997	0.0202	0.0117	0.0555
82.0146	0.0211	0.0108	0.0536	91.9996	0.0219	0.0096	0.0506
83.0016	0.0226	0.0130	0.0616	93.0225	0.0135	0.0112	0.0470
84.0815	0.0194	0.0083	0.0444	94.0100	0.0146	0.0191	0.0720
84.9886	0.0169	0.0098	0.0463	94.9984	0.0143	0.0106	0.0461
85.9619	0.0147	0.0113	0.0487	96.0956	0.0071	0.0102	0.0376
86.9651	0.0147	0.0076	0.0374	97.0023	0.0082	0.0074	0.0303
87.9749	0.0137	0.0083	0.0387	98.0018	0.0232	0.0053	0.0392
88.9867	0.0145	0.0066	0.0343	99.0057	0.0468	0.0030	0.0557

### State identification model verification

Sample data with significant pressure drop changes were extracted from the operation data of the joint station. The PSO-GWO-BP model is utilized for pressure drop prediction. By comparing the deviation and threshold, the boundary between normal and abnormal working areas is clarified to realize the state identification.

**Fig. 12:**
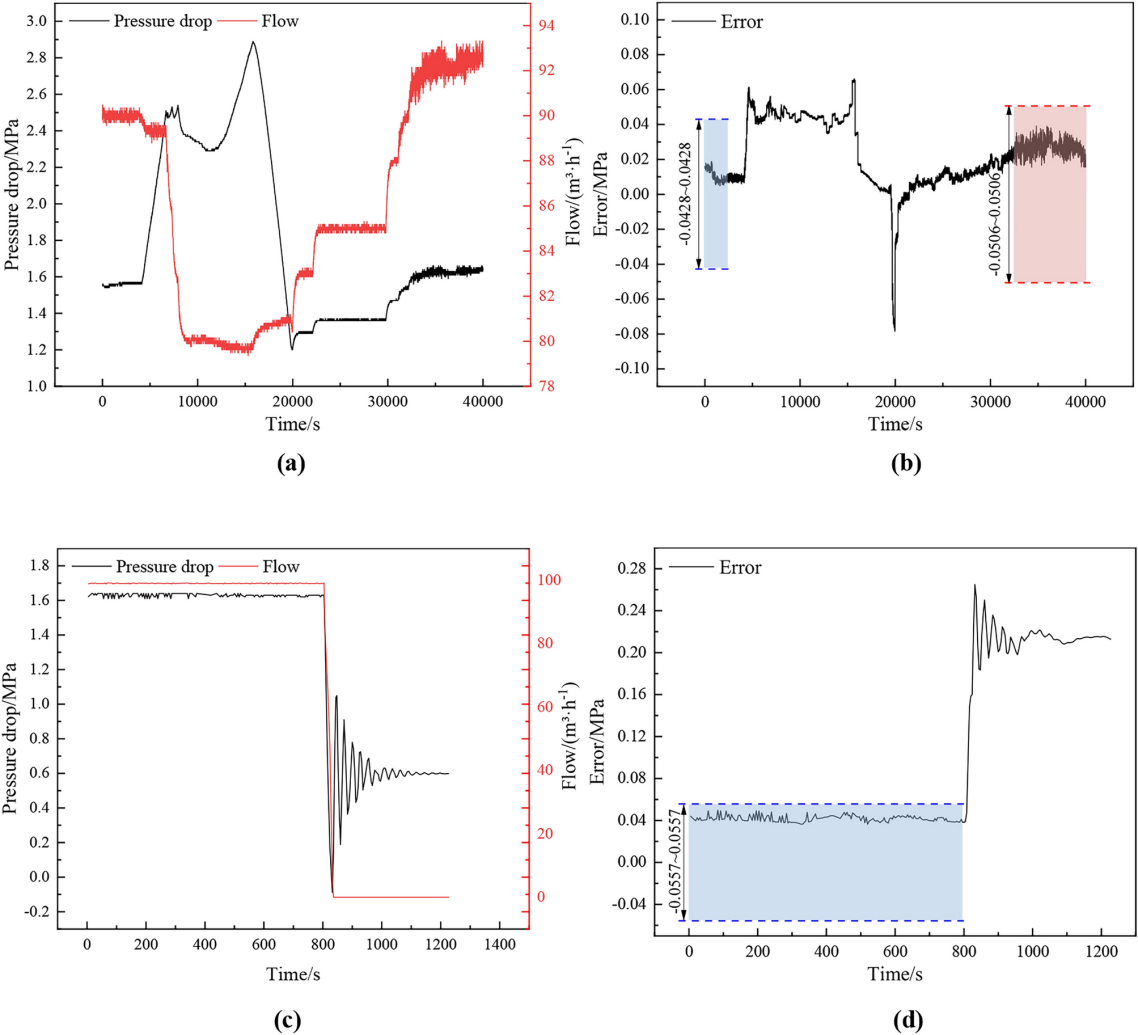
Changes in pressure drop, flow rate and deviation over time: (**a**) and (**c**) are pressure drop and flow rate versus time curves; (**b**) and (**d**) are the deviation versus time curves.

As shown in Fig. 12, (a) and (c) are the curves of pressure drop and flow rate changes at different times, and (b) and (d) are the curves of PSO-GWO-BP predicted values deviated from the measured values. From Fig. 12(a) and (b), it can be observed that the stable flow rates before and after the large change in pressure drop are 90 m^3^/h (blue area) and 92 m^3^/h (red area), corresponding to the thresholds of 0.0428 MPa and 0.0506 MPa, respectively. The deviation of predicted values from measured values is also smaller when the change in pressure drop is small, and all of them are fluctuating in the range of the set thresholds. However, when the pressure drop suddenly increases, the deviation also increases rapidly and exceeds the set threshold. As a result, this working condition is recognized as an abnormal working condition, and then the abnormal working condition recognition module of the intelligent control of the export system is used to further identify the specific types of abnormal working conditions. In this paper, the joint station export system needs to add drag reducing agent when conveying oil. Drag reducers are usually surface-active substances that can change the viscosity and flow characteristics of fluids. By reducing the interaction forces between molecules within the fluid, the drag reducer makes the fluid easier to flow, thereby reducing the friction between the fluid and the pipe wall. The large change in pressure drop in Fig. 12(a) is due to the fact that when the drag reducer was depleted, it was not put in on time due to staff negligence. At this time, the friction between the fluid and the pipeline wall increases, resulting in fluid flow difficulties, thus requiring greater pressure to push the fluid through the pipeline, which leads to a sudden increase in pressure and a flow rate that suddenly reduces; after adding the drag reducer, the pressure and flow rate gradually return to normal operating values. Due to the addition of a drag reducer for the oilfield field operation of the normal, this paper training model selected data are added to the drag reducer operating conditions.

From Fig. 12(c) and (d), it can be seen that the stable flow rate before the large change in pressure drop is 99 m^3^/h, and the corresponding threshold is 0.0557 MPa. When the pressure drop and flow rate are more stable, the export system is in steady-state operation, and the deviation between predicted and measured values is small; when the pressure drop appears to be fluctuating substantially, it rapidly exceeds the set threshold, and the deviation fluctuation changes with the fluctuation of the pressure drop. This trend in pressure drop is due to the occurrence of a dump pump condition, which produces a water hammer effect. When the pump is suddenly and abnormally shut down, the fluid in the piping continues to flow due to inertia, and the sudden change in flow rate leads to large fluctuations in pressure. The kinetic energy of the fluid is instantly converted into pressure energy, forming a pressure wave. The pressure wave will propagate repeatedly in the pipeline and gradually decay until the kinetic energy and pressure energy are rebalanced and the final pressure drop is stabilized near a fixed value. In summary, the state identification model established in this paper can correctly distinguish normal and abnormal working conditions.

## Conclusion

In order to promote the intelligent control of the joint station export system, this paper proposes a PSO-GWO-BP process parameter prediction algorithm and establishes a state identification method based on dynamic thresholds, which realizes the automatic identification of the operating state of the export system. For the PSO-GWO-BP pressure drop prediction model, the advantages of the PSO and GWO algorithms are combined, and by comparing with the THCM model, PSO-BP model, GWO-BP model, POA-BP model, XGBoost model, and CNN model, it is found that the R^2^ of all the models except the THCM model is above 0.91, and the model established in this paper is better than the other models in pressure drop prediction, with R^2^ as high as 0.9877. A scoring mechanism is established based on evaluation indexes to score all models. The PSO-GWO-BP model has the highest score (21), and the CNN model and XGBoost model also show strong ability in pressure drop prediction, ranking second (17) and third (16), respectively. The PSO-GWO-BP model shows the greatest improvement over the THCM model, with an R^2^ increase of 18.2025%, while the R^2^ increase compared to the other models is less than 10%. The intelligent control state identification method of the joint station export system is proposed based on the PSO-GWO-BP model, which utilizes the comparison between the dynamic threshold and deviation to determine whether abnormal working conditions occur. The method is applied to the abnormal working condition data in the production and operation of the joint station to realize the abnormal working condition identification of drag reducer depletion and pump dumping, and the state identification rate reaches 100%. The model developed in this paper exhibits higher accuracy and robustness compared to other models. As the research progresses, future directions can focus on exploring the use of other advanced optimization algorithms in conjunction with the existing model to enhance its global search capability and local optimization performance. In addition, future research could explore the use of more complex neural networks, particularly deep learning models such as Long Short-Term Memory (LSTM) networks. LSTM can effectively handle long-term dependencies in time-series data, allowing it to better capture complex features and improve the accuracy and flexibility of work condition identification. Furthermore, future work could continue to expand the dynamic threshold discrimination method, combining it with other diagnostic indicators (such as flow rate, temperature, etc.) to form a multi-dimensional condition assessment system, which would further enhance the comprehensiveness and accuracy of condition identification.

## Data Availability

The datasets generated and/or analyzed during the current study are available from the corresponding author on reasonable request.
